# GH Therapy in Non–Growth Hormone-Deficient Children

**DOI:** 10.3390/children12010003

**Published:** 2024-12-24

**Authors:** Chiara Guzzetti, Anastasia Ibba, Valeria Incandela, Sandro Loche

**Affiliations:** 1 SSD Pediatric Endocrinology and Neonatal Screening Centre, Microcitemico Pediatric Hospital, 09121 Cagliari, Italy; chiara.guzzetti@aob.it (C.G.); anastasia.ibba@aob.it (A.I.); valeriaincandela@libero.it (V.I.); 2 Research Area for Innovative Therapy in Endocrinology, Bambino Gesù Children Hospital, IRCCS, 00165 Rome, Italy

**Keywords:** children, growth hormone, Turner syndrome, SHOX deficiency, Noonan syndrome, Prader–Willi syndrome, idiopathic short stature, chronic kidney disease, small for gestational age, long-acting growth hormone

## Abstract

Before 1985, growth hormone (GH) was extracted from human pituitaries, and its therapeutic use was limited to children with severe GH deficiency (GHD). The availability of an unlimited amount of recombinant GH (rhGH) allowed for investigating the efficacy of its therapeutic use in a number of conditions other than GHD. Nowadays, patients with Turner syndrome, *SHOX* deficiency, Noonan syndrome, Prader–Willi syndrome, idiopathic short stature, chronic kidney disease, and children born small for gestational age can be treated with rhGH in order to improve adult height. In patients with Prader–Willi syndrome, rhGH therapy also improves body composition and cognitive function. Large post-marketing multinational studies in a large number of pediatric patients demonstrated a good safety profile for rhGH. Recently, long-acting formulations of rhGH have been approved and licensed for GHD, and clinical trials are ongoing for other conditions. In this paper, we review the rhGH therapy in children with conditions other than GHD.

## 1. Introduction

Before 1985, growth hormone (GH) was extracted from human pituitaries, and its therapeutic use was limited to children with severe GH deficiency (GHD). The availability of an unlimited amount of recombinant GH (rhGH) allowed for investigating its efficacy in a number of conditions other than GHD, including Turner syndrome (TS), SHOX deficiency, Noonan syndrome (NS), Prader–Willi syndrome (PWS), children born small for gestational age (SGA), idiopathic short stature (ISS) and chronic kidney disease. A summary of clinical recommendations for rhGH therapy in these patients is reported in Table 1. Recently, long-acting formulations of rhGH (LAGH) have been approved and licensed for GHD, and clinical trials are ongoing for other conditions (Table 2). Large post-marketing multinational studies in tens of thousands of pediatric patients demonstrated, reportedly, a good safety profile for rhGH [1,2,3,4]. However, some adverse events, both in the short and long term, are potentially associated with rhGH therapy and need to be monitored. The most common adverse event in the short term is headache, largely due to intracranial hypertension with pseudotumor cerebri, and is usually transient and benign. Slipped capital femoral epiphysis may also occur. Insulin resistance and the development of malignancies are the most feared adverse events in the long term. Impairment of glucose tolerance and type 2 diabetes mellitus may develop, but the clinical significance appears to be low [5]. Conversely, there is no increased risk of new primary tumors in pediatric patients receiving rhGH therapy [2]. Finally, rhGH therapy affects both thyroxine and cortisol catabolism, which should be monitored before starting treatment and throughout [6,7,8].

In this paper, we review the use of rhGH for the treatment of children with conditions other than GHD.

## 2. Turner Syndrome

TS is a sex chromosome disorder caused by the complete or partial absence or structural abnormalities of one of the X chromosomes. TS is the most common chromosome disorder in females and has a prevalence of 25–50/100,000 [14]. Short stature, hypogonadotropic hypogonadism, and mild skeletal dysplasia are the typical signs in patients with TS, although the clinical presentation is variable. In TS, growth failure may begin during fetal life, and a progressive reduction in height standard deviation score (SDS) may be evident from the age of 12 months [14]. Haploinsufficiency of the short stature homeobox-containing (*SHOX*) gene (pseudoautosomal region (PAR) of the X chromosome) is probably the most important cause of short stature in patients with TS [15], although it is multifactorial. Abnormalities in GH and insulin-like growth factor-1 (IGF-1) function, such as resistance to IGF-1 [16,17,18] and estrogen deficiency [19], may also have a role in the reduction in linear growth. Adult height in untreated girls with TS is approximately 15–20 cm below that of healthy women (144.3 ± 6.7 versus 160.2 ± 6.3 cm) [20].

The use of recombinant rhGH to treat short stature in TS patients was approved almost thirty years ago, and many studies have shown its efficacy on height velocity and adult height [21]. The latest international guidelines [14] recommend starting rhGH therapy early to prevent further loss of potential height. Treatment may be proposed from as early as 2 years of age in the presence of short stature or likelihood of short stature and growth failure (growth velocity below normal value or declining). In case of late diagnosis, rhGH treatment may still be started as long as the epiphyses are open. Treatment should be discontinued when bone age is ≥14 years and/or height velocity is <2 cm/year, as long as there are no physiological reasons for continuing rhGH therapy after epiphyseal closure.

Despite more than fifty studies on rhGH treatment in TS, only a few randomized, controlled trials (RCTs) have compared the treated group with untreated or placebo control groups [22,23,24,25,26], and only two of these RCTs have followed the untreated control group up to adult height [22,24]. Based on these studies, the 2007 Cochrane Center review [21] concluded that the growth velocity of patients with TS treated with rhGH is 2–3 cm/year higher than untreated patients. More recently, a meta-analysis [27] based on data from the two RCTs that followed untreated patients with TS up to adult height [22,24] reported a mean adult height gain of 7.2 cm in patients with TS treated with rhGH, with 40–50% of treated patients that reached an adult height within the normal range, versus only 4–16% of untreated subjects. Other studies reported gains of 15–17 cm in patients with TS treated since a young age with a high dose of rhGH versus estimated adult height [28,29,30]. Large observational studies have confirmed the efficacy of rhGH therapy in TS patients, with an adult height gain of about +1 SDS, but with significant individual variability [3]. In summary, if catch-up growth in the first 2 years of treatment brings height within the normal range and sustained normal height velocity can be achieved, adult height is likely to be in the lower part of the normal range for the majority of patients with TS treated with rhGH. However, these patients may experience absent or minimal pubertal growth spurt with a progressive reduction in the relative prepubertal height SDS gain [31].

The recommended dose of rhGH in patients with TS is 0.045–0.050 mg/kg/day (or 1.3–1.5 mg/m^2^/day) [14] (Table 1). According to international guidelines and local authority-approved dose range, the rhGH dose may be increased to a maximum of 0.068 mg/kg/day (or 2.0 mg/m^2^/day) if the clinical response is suboptimal, the outcome is better in girls with taller target height, younger age at initiation of treatment, and a longer duration between rhGH treatment start and onset of puberty.

Both long-term prospective and observational clinical trials have demonstrated a good safety profile of rhGH treatment in TS [3,32,33,34,35,36,37,38,39,40,41,42,43,44]. However, routine monitoring of IGF-1 concentrations and glucose metabolism is recommended. IGF-1 should be maintained within the normal range for age, sex, and pubertal stage. If IGF-1 levels are persistently higher than the normal levels for age, rhGH dose reduction is recommended [14]. Girls with TS appear to have an increased risk of intracranial hypertension, slipped capital femoral epiphysis, and scoliosis during rhGH treatment compared with patients with idiopathic GHD or ISS [3,45,46]. However, scoliosis is common in girls with TS regardless of rhGH therapy [47]. Likewise, girls with TS have a higher risk of carbohydrate metabolism impairment [48,49,50], and transient abnormalities of carbohydrate metabolism have been reported during or following rhGH therapy [29,51,52,53]. Finally, there is no evidence of a higher risk of neoplasm in patients with TS treated with rhGH [14].

Recently, LAGH has been approved for the treatment of patients with GHD (Table 2). Several trials with LAGH in children with TS are ongoing [9]. A single 2-year retrospective study [10] showed that pegylated LAGH has a comparable effect to daily rhGH therapy on growth promotion, and no serious adverse effects have been reported. Further studies are required to recommend LAGH formulations for the treatment of short stature in patients with TS.

Other therapies have been investigated to improve stature in TS patients. Off-label use of oxandrolone combined with rhGH has been reported to increase adult height (2–4 cm) [54,55]. However, the FDA withdrew oxandrolone in 2023 due to adverse events, and accordingly, the latest guidelines no longer recommend its use in TS [14].

Other authors speculated that the use of ultra-low-dose oral ethinyl estradiol during the prepubertal period, combined with rhGH, could increase adult height [21,25,56]. However, further studies are required to confirm long-term growth benefits and establish the formulation, route, and dosing of estrogen. Again, the latest guidelines do not recommend the use of prepubertal, very low-dose estrogen replacement as a growth-promoting therapy in patients with TS [14].

Finally, low-dose estrogen replacement should be started between 11 and 12 years of age if pubertal induction is required (usually for high FSH on two consequent evaluations) [14]. There is no sufficient evidence that delaying pubertal induction (at about 14 years old) results in a taller adult height.

## 3. *SHOX* Deficiency

The *SHOX* gene deficiency is the most common monogenic cause of growth abnormalities and may be responsible for isolated or syndromic short stature [57]. The *SHOX* gene, located in the region PAR-1 of the short arms of both sex chromosomes, acts in a dose-dependent way, and both males and females must express two active copies of this gene. Thus, haploinsufficiency or complete loss of function of *SHOX* determines abnormal proliferation and differentiation of chondrocytes with compromised growth of the long bones. The phenotype varies from mild isolated short stature (loss of function of one allele) to more extreme phenotype such as Leri–Weill syndrome (severe short stature, abnormal craniofacial phenotype, and mesomelia of the upper and lower limbs) in the case of haploinsufficiency, and Langer syndrome osteodysplasia (extreme short stature, mesomelia, and limb deformity) in the case of complete loss of function of *SHOX* gene. Conversely, expressing an extra copy of *SHOX* can cause tall stature, as is seen in Klinefelter syndrome (47, XXY) [58,59,60].

Children with *SHOX* mutations have been shown to benefit from rhGH therapy (Table 1), with outcomes similar to girls with TS [59,60]. To our knowledge, only one RCT evaluating GH therapy in *SHOX* deficiency has been published [59].

Data from the multicentric observational “Genetics and Neuroendocrinology of Short Stature International Study” (GeNeSIS) [61] confirm the efficacy and safety of rhGH therapy in these patients. A more recent study [62] reported that the adult height of patients with *SHOX* haploinsufficiency treated with rhGH therapy was 6.3 cm (1 SD) higher than untreated ones. In the same study, the authors reported that the use of GnRH analogs in addition to rhGH in pubertal patients allowed the attainment of an adult height similar to patients who started rhGH therapy alone in the prepubertal age. Further studies are needed to confirm these data.

## 4. Noonan Syndrome

NS is an autosomal dominant disorder with an estimated incidence of 1 in 1.000–2.500 live births. The phenotype is variable, with 50–70% of patients presenting short stature. Other features may include early feeding difficulties and mild developmental delay, typical facial features such as hypertelorism, large head and forehead, down slanting eyes, epicanthal fold, heart defects (stenosis of the pulmonary valve or hypertrophic cardiomyopathy), and chest and spinal deformities [63,64].

NS is caused by mutations in genes that encode proteins of the RAS-MAPK signal transduction pathway [64,65]. A number of genes have been described associated with NS. For most genetic mutations, the transmission follows a dominant pattern of inheritance (PTPN11, BRAF, KRAS, MAP2K1, MRAS, NRAS, RAF1, RASA2, RIT1, RRAS2, SOS1, or *SOS2* [66]). Both dominant and recessive mutations have been described for *LZTR1* [67,68].

The use of rhGH treatment in patients with NS was first approved by the FDA in 2007 and then almost worldwide. Although some studies have reported reduced response to rhGH treatment in PTPN11-positive patients [69,70], the majority of them have demonstrated an increased growth velocity and an increased mean height SDS [71,72,73,74,75,76], with a mean height gain of 0.6–2.0 SDS. The outcome is significantly correlated to the dose, age at the start, bone age, and the duration of treatment, but not gender [71,73]. No RTCs have been carried out to date in patients with Noonan syndrome.

The suggested dose varies among studies, ranging from 0.035 to 0.066 mg/kg daily [71,72,77] (Table 1). No severe adverse events have been reported [73]. The RAS-MAPK pathway is important for cellular differentiation and proliferation, and mutations of its components increase the risk of malignancy in patients with NS regardless of the use of rhGH therapy [78,79,80,81]. Although it is not known whether rhGH treatment may affect cancer risk, close monitoring of IGF-1 levels is highly recommended [82]. A phase III study is ongoing to compare the efficacy and safety of weekly rhGH in children with NS [83] (Table 2).

## 5. Prader–Willi Syndrome

PWS is a complex, rare disease with an incidence of approximately 1 in 16.000 to 1 in 21.000 live births. PWS is an imprinting disorder caused by three main mechanisms affecting the genes that are exclusively expressed in the paternally inherited chromosome, specifically in the 15q11.2–q13 region [84]. Most cases (61%) are due to paternal deletion of the 15q11.2–q13 region, followed by maternal uniparental disomy (UPD in which both chromosome 15 are inherited from the mother) in the 36% of the cases, and an imprinting defect (ID, 3%) [85].

The phenotype differs according to age. Newborns may present severe hypotonia and feeding problems, while children may present developmental delay and hypothalamic hyperphagia, which lead to a progressive development of obesity associated with temper tantrums [86,87]. Short stature, adrenal insufficiency [88], hypogonadism [89], and hypothyroidism may also be associated.

RhGH therapy has changed the natural history of PWS [90]. A number of studies, including RCTs [87,91,92,93,94], have shown the effectiveness of rhGH in improving growth velocity and stature, body composition, lipid profile, exercise tolerance [95], psychomotor development [91,96,97,98,99], muscle strength, bone health [100], and preventing complications of PWS patients, subsequently increasing their life span [91]. Early treatment is associated with better improvements in psychomotor development [96,101].

RhGH should be started before 1 year of age, or at least as soon as possible before the onset of obesity [102], and continued for as long as the benefits outweigh the risks [103]. Severe obesity, uncontrolled diabetes, untreated severe obstructive sleep apnea, active cancer, and active psychosis must be ruled out before starting treatment [103].

Current guidelines [103] suggest starting with a daily dose of 0.5 mg/m^2^/day to a maximum of 1.4 mg/m^2^ (~0.047 mg/kg) according to the clinical response and IGF-1 levels that should always remain within the normal range (Table 1). No major adverse events have been reported during rhGH treatment in PWS, and no increased risk of diabetes, scoliosis, or central and obstructive apnoea have been observed [91,102,104,105,106]. Reports of sudden infant death in infants with PWS have raised concerns about the possible effect of rGH therapy in this population, possibly related to tonsillar/adenoid hypertrophy [107]. However, studies of causes of death in individuals with PWS have found no differences in the prevalence of sudden death between those receiving and not receiving GH treatment [108]. A recent systematic review reported the outcomes of rGH therapy in the obstructive sleep apnoea parameters and concluded that none of the studies demonstrated statistically significant modifications of Prader–Willi patients related to the growth hormone administration [109]. Some authors reported a higher risk of leukaemia and lymphoma not related to rhGH in patients with PWS [110].

## 6. Small for Gestational Age

Children born SGA have birth weight and/or birth length below −2 SDS for their gestational age. In the first 2–3 years of life, most of them catch up with growth (defined as a growth rate higher than 0 SDS, allowing them to reach a height into the normal height range, according to the mid-parental height). However, about 10% of SGA children will have persistent short stature and are at risk of becoming short adults [111,112,113].

Several studies, including one RCT [114], have shown that rhGH therapy is effective in increasing adult height in these patients [115,116,117,118,119,120], with an increase in mean height gain of +1.25 SDS, although variable between different studies. RhGH therapy has been approved since 2001 for children born SGA without catch-up growth. RhGH therapy-induced catch-up growth is characterized by normal body proportions [116,121]. The recent international consensus guidelines [113] recommend starting rhGH therapy in short children born SGA at 3–4 years of age when it is unlikely that catch-up growth may occur spontaneously after having ruled out other causes for short stature.

The recommended rhGH starting dose is 0.033 mg/kg/day, to be increased to a maximum dose of 0.067 mg/kg/day if growth response is unsatisfactory (height gain < 0.5 SDS) during the first year of treatment (Table 1). It is recommended that IGF-1 concentrations be monitored at least annually.

A number of factors can be associated with growth response to rhGH treatment in children born SGA [114,119,122,123,124,125], and among them, duration of treatment, age, height at rhGH start, birth length, and birth height are the most important. The variability in response to rhGH therapy is likely to be also associated with possible underlying genetic variants responsible for intrauterine growth restriction and short stature [126]. RhGH therapy is not suggested in genetic disorders with a predisposition to develop tumors (i.e., DNA repair disorders, chromosomal breakage syndromes, and others) [113].

RhGH treatment has proven to be safe in this population [4]. SGA children commonly have reduced insulin sensitivity, which may worsen during rhGH treatment [127]. However, glycated hemoglobin concentrations remain within the normal range, and type 2 diabetes mellitus does not develop [128,129,130]. This phenomenon is probably due to a compensatory increase in insulin secretion owing to the insulin-antagonistic effects of GH [128,129,131,132]. Insulin sensitivity improves after rhGH cessation [114,131]. According to recent guidelines [113], during rhGH treatment, it is recommended that thyroid function is monitored at least annually. Routine evaluation of markers of lipid and glucose metabolism (fasting serum lipid, glucose, insulin, and HbA1c levels) is recommended only for SGA children with associated risk factors like overweight or obesity or family predisposition.

Recently, LAGH resulted in effective and safe results in SGA patients in the first phase 2 study after 52 weeks of treatment (Table 2). This study has shown that treatment with somapacitan at the dose of 0.24 mg/kg/week was not inferior to daily rhGH at the dose of 0.067 mg/kg/day in children born SGA [12].

## 7. Idiopathic Short Stature

ISS is defined as a condition with height SDS ≤ −2.0 for a given age, sex, and population group, associated with growth rates unlikely to permit the attainment of adult height in the normal range, with normal birth height and weight, without evidence of systemic, nutritional, endocrine, or chromosomal abnormalities. This definition includes a heterogeneous group of children with unidentified causes of short stature, as well as short children with constitutional delay of growth and puberty and familial short stature [133].

The first short-term trial in children with ISS treated with pituitary-derived GH was conducted in 1983. The authors reported an increase in growth rate of 2 cm/year during GH therapy [134]. Since then, a large number of studies have been published [135,136,137], but only a few of them are controlled and/or randomized [138,139,140]. A recent metanalysis [141], including both the controlled (115 children, 79 cases, and 36 controls) [138,139,140] and non-controlled studies (477 children, 181 cases, and 296 controls) [142,143,144,145,146,147,148], reported a mean adult height gain of 0.45–0.65 SD (about 3–4 cm). The authors concluded that no single, high-quality evidence trial was conducted up to adult height, that the overall effect of rhGH therapy in ISS patients was lower than in other conditions, and that the response to therapy was extremely variable among studies [141]. Many factors are involved in the individual variability in response to rhGH therapy, including age at the start of therapy, length at birth, the dose of GH, the difference between height and target height, and the difference between age and bone age [149].

In 2003, the FDA approved rhGH therapy for ISS children, while EMA has not authorized this treatment. Indeed, to date, the real efficacy of rhGH treatment in patients with ISS is still debated [137].

Thus, current guidelines [2] suggest making the decision to treat with rhGH on an individual basis after an assessment of physical and psychological burdens and a discussion of risks and benefits with the parents. The recommended initial dose is 0.24 mg/kg/week, to be titrated according to clinical response to a maximum dose of 0.47 mg/kg/week (Table 1). Discontinuation of rhGH therapy is recommended if the response after one year of treatment is not adequate (increase in height SDS > 0.3–0.5).

Aromatase inhibitors have been used to delay bone maturation alone or in combination with rhGH [150,151,152,153]. However, the use of aromatase inhibitors is still not approved due to potential adverse effects (increased insulin resistance, reduced high-density lipoprotein cholesterol, vertebral deformities, long-term effects on spermatogenesis and infertility, and impairment of cognitive function) [154]. Nevertheless, a recent meta-analysis on seven RCTs provides reassuring data on the efficacy and safety of letrozole in constitutional delay in growth and puberty [155].

Finally, studies are ongoing to evaluate the efficacy, safety, and tolerability of LAGH formulation in ISS children, with promising results [13] (Table 2).

## 8. Chronic Kidney Disease

Most patients with chronic kidney disease (CKD) have decreased growth rates with consequent short stature, which is often not normalized by dialysis and results in reduced adult height [156]. A large number of factors are responsible for the growth failure observed in these children. Among them, the most important factors are malnutrition, abnormalities of the GH/IGF-1 axis with GH insensitivity, and corticosteroid therapy [157]. A number of trials, including RCTs, have shown that rhGH therapy increases growth rate and improves height SDS in children with chronic renal failure [158,159]. Current guidelines recommend that children aged above 6 months on dialysis or with stages 3–5 of CKD should be candidates for rhGH therapy if they have persistent growth failure (height < 3° percentile for age and sex and height velocity < 25° percentile, or height between 3° and 10° percentile and persistent height velocity < 25° percentile) once other potentially treatable causes of growth failure have been considered. Children who received a kidney transplant can be treated with rhGH if they have persistent growth failure 1 year after transplantation and if steroid-free immunosuppression is not possible. In children with persistent growth failure due to nephropathic cystinosis, rhGH therapy may be proposed at all stages of CKD.

RhGH therapy should not be started in patients with severe secondary hyperparathyroidism (parathyroid hormone > 500 pg/mL), diabetic retinopathy, acute critical illness, and active malignancy [156].

The recommended dose of rhGH is 0.045–0.05 mg/kg/day [156] (Table 1). The response to rhGH therapy should be regularly assessed in terms of both efficacy and safety, with adjustments to the rhGH dose or eventual discontinuation in case of adverse events. Poor response to rhGH therapy is common in children with CKD. According to the Summary of Expert Opinion [157], rhGH should be discontinued if height velocity is below 2 cm/year after 1 year of therapy.

The expected height gain after 2–5 years of rhGH treatment is estimated to be about 7 cm in prepubertal children [156,158], whereas in prepuberty, there is no convincing evidence [160,161].

A number of open issues are still in place on the use of hGH in patients with CKD. Among all, the effect on adult height is poor and unpredictable [157] due to the extreme heterogeneity of the patients. In fact, CKD has multiple causes, and its duration is highly variable. Associated steroid therapy, nutrition, hormone resistance, and comorbidities are among other confounding factors. For these reasons, a personalized approach to the patient is of paramount importance, and the option to start GH treatment should be carefully evaluated for every single patient [162].

## 9. Conclusions

RhGH treatment is classically indicated in patients with GHD and has proven effective and safe. RhGH treatment produces an increase in growth velocity and normalizes adult height in a great number of SGA children. Although, to a lesser extent, rhGH therapy is also effective in increasing adult height in girls with Turner syndrome, *SHOX* deficiency, Noonan syndrome, and children with chronic kidney disease. RhGH therapy has also proven beneficial in patients with Prader–Willi syndrome, with positive effects on growth, body composition, muscle function, and cognition. RhGH therapy has been approved in many countries also for children with ISS, although its efficacy is still debated.

In all conditions, the response to therapy exhibits a large interindividual variability and is difficult to predict. Therefore, the decision to start GH treatment must be considered on an individual basis, taking into account auxological, clinical, and psychosocial issues.

In the near future, LAGH formulations will probably be available to treat these patients and hopefully will improve their adherence to therapy and quality of life.

## Figures and Tables

**Table 1:** Recommendations for rhGH therapy in conditions other than GHD.

	rhGH Dose	Criteria for rhGH Therapy	Approved by
**Turner syndrome**	0.045–0.068 mg/kg/day	Short stature and/or growth failure	FDA and EMA
***SHOX* deficiency**	0.045–0.050 mg/kg/day	Short stature	FDA and EMA
**Noonan syndrome**	0.035–0.066 mg/kg/day	Short stature	FDA and EMA
**Prader–Willi syndrome**	0.5–1.4 mg/m^2^/day	Absence of severe obesity, uncontrolled diabetes, untreated severe obstructive sleep apnea, active cancer, and active psychosis	FDA and EMA
**Small for gestational age**	0.033–0.067 mg/kg/day	Short stature Age > 3–4 years	FDA and EMA
**Idiopathic short stature**	0.24–0.47 mg/kg/week	Short stature	FDA
**Chronic kidney disease**	0.045–0.05 mg/kg/day	Short stature and/or growth failure Age > 6 months Dialysis or stage 3–5 of CKD or kidney transplant or nephropathic cystinosis Absence of severe secondary hyperparathyroidism, diabetic retinopathy, acute critical illness, and active cancer	FDA and EMA

**Table 2:** Clinical trials on LAGH in conditions other than GHD.

	Published	Ongoing [9]
**Turner syndrome**	Gao et al., 2022 [10] Jintrolong, 75 patients, 2 years of therapy	NCT05690386, Lonapegsomatropin NCT05330325, Somapacitan NCT05723835, Somapacitan NCT05838885, YPEG-rhGH
***SHOX* deficiency**	-	-
**Noonan syndrome**	-	NCT05330325, Somapacitan NCT05723835, Somapacitan
**Prader–Willi syndrome**	-	-
**Small for gestational age**	Juul et al., 2023 [11] Somapacitan, 62 patients, 26 weeks of therapy Juul et al., 2024 [12] Somapacitan, 62 patients, 52 weeks of therapy	NCT05330325, Somapacitan NCT05723835, Somapacitan NCT03878446, Somapacitan NCT05838885, YPEG-rhGH
**Idiopathic short stature**	Choi et al. [13] EutropinPlus, 10 patients, 12 months of therapy	NCT05330325, Somapacitan NCT05723835, Somapacitan NCT05838885, YPEG-rhGH
**Chronic kidney disease**	-	-
